# Climate and Soil Properties Drive the Distribution of Minor and Trace Elements in Forest Soils of the Winter Olympic Core Area

**DOI:** 10.3390/biology14010082

**Published:** 2025-01-16

**Authors:** Xiaochang Wu, Huayong Zhang, Zhongyu Wang, Wang Tian, Zhao Liu

**Affiliations:** 1 Research Center for Engineering Ecology and Nonlinear Science, North China Electric Power University, Beijing 102206, China; 2 Theoretical Ecology and Engineering Ecology Research Group, School of Life Sciences, Shandong University, Qingdao 266237, China

**Keywords:** soil minor and trace elements, climate, soil properties, the core area of the Winter Olympics

## Abstract

Minor and trace elements in soil play a crucial role in regulating ecological processes that sustain the functionality of forest ecosystems. In this study, we have selected three conifer forests (*Pinus sylvestris*, *Picea asperata*, *Larix principis-rupprechtii*), one broadleaf forest (*Betula Platyfilla*) and one mixed forest of *Betula Platyfilla* and *Larix principis-rupprechtii* in the Winter Olympic core area and determined the pattern of 12 typical elements (B, Fe, V, Cr, Ni, Co, Mn, As, Cu, Zn, Sn and Se) in soils and their main drivers in the three different soil layers (A, B and C horizon) in each soil profile. Our results showed that the concentrations of B, Fe, Cr, Cu, Ni and Sn were mainly enriched in the broadleaf forest and mixed broadleaf–conifer forest zones, and the average concentrations of Co, Mn, V, Zn, As and Se were mainly enriched in coniferous forest zones in contrast. We have observed that the mean concentrations of Fe, Cr, Ni, Zn, As, Sn and Co increase with soil depth in the BP forest. The concentrations of Se and Cu were higher in the A layer than the C layer. The piecewise structural equation modeling (piecewiseSEM) results visualized a direct and negative effect on B, Fe, V, Cr and Ni concentrations due to soil temperature, while the concentrations of Se is mainly influenced by soil temperature and soil properties.

## Simple Summary

Minor and trace elements are found in the environment in extremely small quantities but have powerful bioscience roles, and they may cause hazardous effects when levels exceed certain limits. Consequently, it is essential to ascertain the concentrations of these elements within the soil. This paper addresses the distribution of 12 minor and trace elements in five different forests and soil depths in the Winter Olympic core area and explores the mechanisms driving element distribution. The results of this study indicate that soil minor and trace elements are mainly influenced by climatic factors and soil properties, and no direct effect of vegetation type on soil element distribution was observed in this study. Understanding the distribution of minor and trace elements in forest soils across the core area can provide important information on soil succession patterns in the boreal forest and may help elucidate the response of elemental distributions to climate change.

## 1. Introduction

Boreal forests represent a significant element of terrestrial ecosystems [1], comprising approximately one-third of the world’s forested area and contributing 20% to the global carbon sink [2]. Boreal forests have significant economic benefits and cultural value to society [3,4]. Climate change and human activities are known to affect boreal forest ecosystems [5,6], which greatly affect the structure and ecological processes of boreal forests [3]. The core area of the Winter Olympics is characterized by forest vegetation, with ecological succession predominantly involving *Betula Platyfilla*. In addition, the area is planted with a large number of coniferous forests such as *Pinus sylvestris*, *Picea asperata*, *Larix principis-rupprechtii*, which are mixed to varying degrees with *Betula Platyfilla* to form mixed broadleaf–conifer forest. As the world’s largest comprehensive winter games, the Winter Olympics will cause natural forest disturbances to the local ecosystem from the planning of the competition area to the construction of the venues. These increased disturbances would pose growing pressures on boreal forests.

The most important characteristic of minor and trace elements is their very low concentration in the environment [7]. They include a variety of chemical groups—metals (e.g., Fe, Cu, and Zn), metalloids (As) and non-metals (Se)—which vary in their physicochemical properties in the environment [8]. The minor and trace elements have powerful bioscientific effects and may cause harm when levels exceed certain limits [9,10]. Some of these trace elements are required for many biological activities (e.g., Fe, Cu and Zn), for example, for the transport of oxygen, protein synthesis and enzyme activities [11]; others such as Cr, Co and B may replace essential metals, and they can disrupt the proper functioning of cofactors and enzymes, which can lead to toxic effects in organisms [12]. However, even the former metal can have toxic effects if the concentration is elevated [12,13]. The core area of the Winter Olympics also undertakes the important function of water conservation in the Beijing–Tianjin–Hebei region, Consequently, it is essential to ascertain the concentrations of these elements within the soil.

The biogeochemical cycling of elements is influenced by soil matrix characteristics and soil weathering processes [14]. These cycles are further regulated by the activities of vegetation and microorganisms [15,16] and are also affected by climatic conditions [17,18,19]. Climate is the most dominant factor influencing the spatial distribution of elements [20]. Temperature further influences trace element distribution [21] by regulating plant growth and microbial activity [22]. Soil moisture plays a crucial role in the formation and evolution of various soil types and the transport and transformation of trace elements [23]. An analysis of 9830 soil samples from southeastern China revealed spatial variability in the concentrations of 53 elements related to temperature, precipitation and pH [24]. Patterns of the lowest soil element (Cu, Fe, Mn, Zn and Ni) concentrations were revealed within arid and tropical ecosystems globally. In addition, a temperature threshold of 12–14 °C was identified, above which all soil minor and trace elements are abruptly reduced [25]. The Winter Olympic core area is known for its long, bitter winters, short summers, and snowfall, which makes up the majority of the yearly precipitation. The fundamental factors contributing to the fluctuations in the concentrations of various minor and trace elements remain largely unidentified. Comprehending the influence of environmental gradients on variations in elemental concentrations could provide insights into the sudden alterations in ecological responses to these elements [26,27].

We have selected five forests, *Pinus sylvestris* (PS), *Picea asperata* (PA), *Larix principis-rupprechtii* (LP), *Betula Platyfilla* (BP) and the mixed forest of *Betula Platyfilla* and *Larix principis-rupprechtii* (MF), to investigate the distribution characteristics of soil minor and trace elements in the Winter Olympic core area. We specifically focus on the forest type and soil depths. Additionally, we aim to investigate how environmental factors affect soil trace and minor elements by piecewise structural equation modeling (piecewiseSEM). Our specific objectives of this research are (1) to determine the distribution of soil minor and trace element concentrations in the Winter Olympic core area and (2) to identify the main drivers affecting trace element distribution in the Winter Olympic core area.

## 2. Materials and Methods

### 2.1. Study Area

The 118 km^2^ Winter Olympic core area is located in Hebei Province, China, about 22 km northeast of Chongli District (40°47′ N to 41°17′ N and 114°17′ E to 115°34′ E). This region, which is between 1797 and 2003 m above sea level, has a typical East Asian continental monsoon climate, with cold winters and cool, humid summers. With yearly precipitation of 483.3 mm and average temperatures between 3.7 and 19 °C, the climate is categorized as continental monsoon. As of 2021, the forest coverage rate in the region is reported to be 67%, characterized by a relatively simplistic forest structure. The forests of Chongli are home to a total of 553 species of terrestrial wild plants, which are classified into 301 genera across 80 families.

### 2.2. Experimental Design

We conducted sampling in five forest types, including *Pinus sylvestris* (PS), *Picea asperata* (PA), *Larix principis-rupprechtii* (LP), *Betula Platyfilla* (BP) and the mixed forest of *Betula Platyfilla* and *Larix principis-rupprechtii* (MF). The sampling was carried out in July 2019. Three samples of each type of forest were selected in order from top to bottom along the direction of runoff. Each profile was categorized into three distinct layers of soil formation: A (surface horizon, characterized by a darker color), B (granular with a prismatic structure) and C (bottom layer consisting of unconsolidated earth material). In each layer, samples of ring knives were collected, and composite soil samples were subsequently analyzed to assess their physical and chemical properties. Since these forests were distributed in close proximity and there were no significant differences in mean annual temperature and annual precipitation conditions, we used mean annual soil temperature (ST) and mean annual soil humidity (SH) instead of temperature and precipitation (Table S1).

### 2.3. Soil Sample Collection and Chemical Analyses

Following the removal of litter and other debris from the soil surface, a soil profile measuring one meter in depth was excavated using a spade. Soil samples were then obtained utilizing a ring knife. Two samples from each plot were collected with the ring knife, and the composite samples were subsequently analyzed to assess the chemical and physical properties of the soil.

The soil was subjected to sieving using a 2 mm mesh. The bulk density (BD) of the soil was assessed by drying the sample in an oven at 105 °C for a duration of 48 h or until a consistent weight was obtained. The bulk density was subsequently calculated by dividing the weight of the oven-dried soil by its volume. Total porosity was assessed by measuring the weight of soil that had absorbed an adequate amount of water to reach saturation, which was calculated by taking the difference between the weight of the saturated soil and the weight of the oven-dried soil and then dividing this value by the volume of the soil. Soil pH was assessed utilizing a pH meter (type: PHS-3C by Shjingmi, Shanghai, China) employing a soil-to-water ratio of 2.5:1. Soil organic matter (SOM) was determined by the external heating method of potassium dichromate and concentrated sulfuric acid [28]. Purging and trapping methodologies were employed to ascertain the concentration of alkali-hydrolyzable nitrogen (AHN) utilizing an elemental analyzer (type: Elementar Vario Macro cube by Elementar, Frankfurt, Germany). The concentrations of available phosphorus (AP) [29] was quantified using inductively coupled plasma–optical emission spectrometry (type: Agilent 5110 ICP–OES by Agilent Technologies, Santa Clara, CA, USA). Soil samples were digested using aqua regia (3HCl + HNO_3_). Two blank controls, duplicate samples and a standard reference material (GBW07404 by IGGE, Langfang, Hebei, China) in each batch were simultaneously digested to ensure quality control. The concentrations of V, Mn, Fe, Co, B and Sn were determined by inductively coupled plasma–mass spectrometry (ICP-MS, Agilent 5110 by Agilent Technologies, Santa Clara, CA, USA), while Cr, Ni, Cu, As and Zn were measured by atomic fluorescence spectrometry (240AFS AA by Agilent Technologies, Santa Clara, CA, USA). Additionally, Se concentrations were assessed using atomic fluorescence spectrometry (AFS-930, Beijing, China).

### 2.4. Statistical Analysis

The data presented in this study were characterized using the mean and standard deviation. The concentrations of SOM, AHN and AP were described by mass content. Normalization of data prior to analysis using Z-Score. Analysis of variance (ANOVA) and Duncan’s test were employed to systematically assess variations in soil chemical and physical characteristics and the distribution of soil elements across different soil horizons and forest types. Statistical significance was established at a threshold of *p* < 0.05. Pearson correlation analysis and principal component analysis (PCA) were conducted to investigate the relationships among various concentrations of elements and the associated environmental factors. The classification of 12 elements using PCA will help to further explore the mechanisms by which climate and soil properties drive soil elemental distributions. Using the KOM spherical test, the principal components with eigenvalues greater than 1 are selected according to the eigenvalue size order, and the elemental loadings in each principal component are obtained by rotating the matrix. The initial four principal components (PC1, PC2, PC3, and PC4) derived from the principal component analysis (PCA) explained 81.2% of the variance associated with soil elements, thereby successfully representing the variability in the concentration levels of all twelve minor and trace elements analyzed.

Subsequently, the piecewise structural equation modeling (piecewiseSEM) approach was used to explore the mechanisms of soil minor and trace elements in response to environmental factors. This modeling approach effectively and clearly illustrates both the direct and indirect relationships between essential ecological factors and their impacts on soil components. Based on the existing theoretical framework research progress, several variables were selected for inclusion in the model. The piecewise SEM analysis was conducted utilizing the “piecewiseSEM” package [30]. We employed Fisher’s C test to assess the adequacy of the model fitting results [31]. The models underwent iterative modifications and refinements, guided by the statistical significance of the pathways (*p* < 0.05) and the overall goodness of fit (0 ≤ Fisher’s C/df ≤ 2 and 0.05 < *p* ≤ 1.00).

All statistical analyses were performed utilizing SPSS 25.0 Software (IBM, Armonk, NY, USA) and R 4.0.5 (R Development Core Team 2021 R Foundation for Statistical Vienna, Austria).

## 3. Results

### 3.1. Difference in Concentrations of Minor and Trace Elements in Five Forest Soils

**Figure 1:**
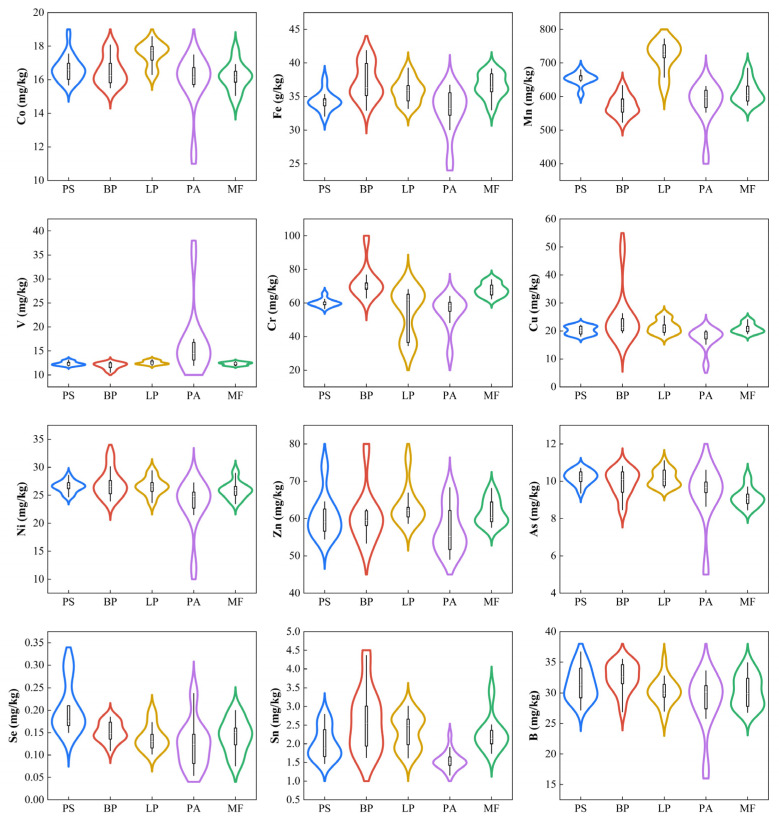
Violin plot of soil minor and trace elements with boxes in five forests.

The concentrations of minor and trace elements in the soils predominantly exhibited a positively skewed or bimodal distribution pattern (Figure 1). Various coefficients of variation (CV) (Table S3) for elements in different forests showed great variation, indicating significant heterogeneity. The mean concentrations of B, Fe, Cr, Cu, Ni and Sn in the five forests were highest in BP followed by LP and MF. The average concentrations of Co, Mn and Zn were highest in LP, and V was highest in PA. PS soils present higher As and Se contents; however, these levels remain within a comparable range. The average concentrations of V and Se were similar in all five forests, but the high CVs (V: 42%, Se: 42%) in PA indicated high spatial variability.

### 3.2. Influences of Soil Depth and Forest Types on Minor and Trace Elements Concentrations

**Figure 2:**
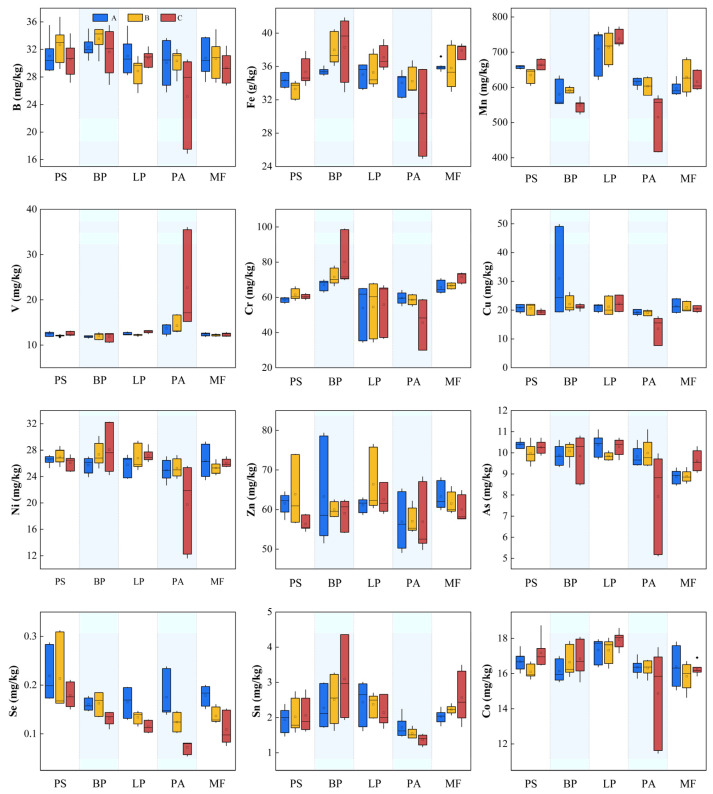
Vertical distribution of soil minor and trace elements in different layers among five forests.

The concentrations of all tested elements in five forest soils exhibited remarkable spatial variability with soil depth (Figure 2). The mean concentrations of Fe, Cr, Ni, Zn, As, Sn and Co increase with soil depth in the BP forest. BP soils had the highest Fe, Cr, Ni and Sn concentrations. There was no significant trend in concentrations of Zn, As and Co among all five forests.

The B concentrations present a trend of increasing then decreasing with soil depth in PS, BP, PA and MF but a trend of decreasing then increasing in LP. The concentrations of Cu decrease with increasing soil depth in PS, PA and MF forests. The concentrations of Se were higher in the A layer than the C layer among all five forests, and PS soil had the highest Se concentrations. There was no significant trend between soil layers for Mn and V concentrations. LP forest soils had the highest Mn concentrations, while PA present much higher soil V concentrations than those in other forests.

### 3.3. Relationships of Soil Minor and Trace Elements with Climate Soil Proportions

**Figure 3:**
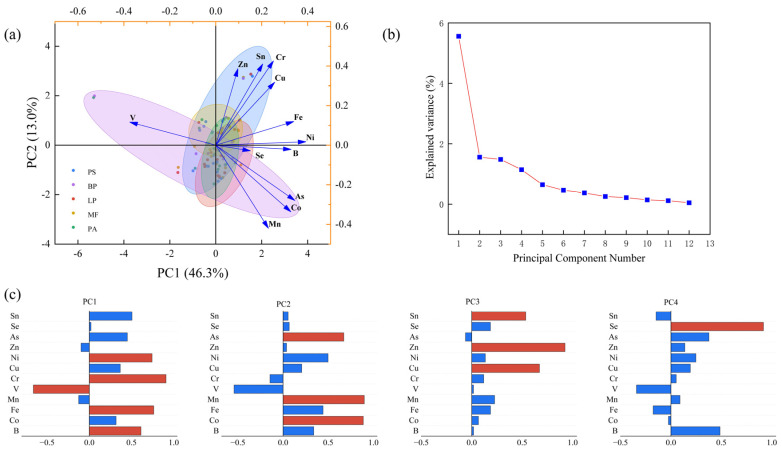
Principal component analysis of minor and trace elements in five forests. (**a**) Loading plot, (**b**) variance explained. (**c**) Bar plots illustrating the loadings of each minor or trace element. The orange bars denote the loadings and contributions that are deemed significant.

Based on the findings from the principal component analysis (Figure 3a), we categorized the 12 measured elements into four principal components by analyzing the explained variance associated (Figure 3b) with each component, as well as the loadings of minor and trace elements and micronutrients (Figure 3c). PC1 explained 46.3% of the total variance and exhibited a significant correlation with the concentrations of B, Fe, V, Cr and Ni contents; these can be categorized as siderophile elements. PC2 accounted for 13% of the variance observed in the original dataset, with Co, Mn and As exhibiting the most significant loadings. PC3 accounted for 12.4% of the total variance, correlated to Cu, Zn and Sn contents. PC4 explained 9.5% of the variance, while the concentrations of Se mainly explained. The initial four principal components derived from the PCA (PC1, PC2, PC3 and PC4) collectively explained 81.2% of the total variance.

**Figure 4:**
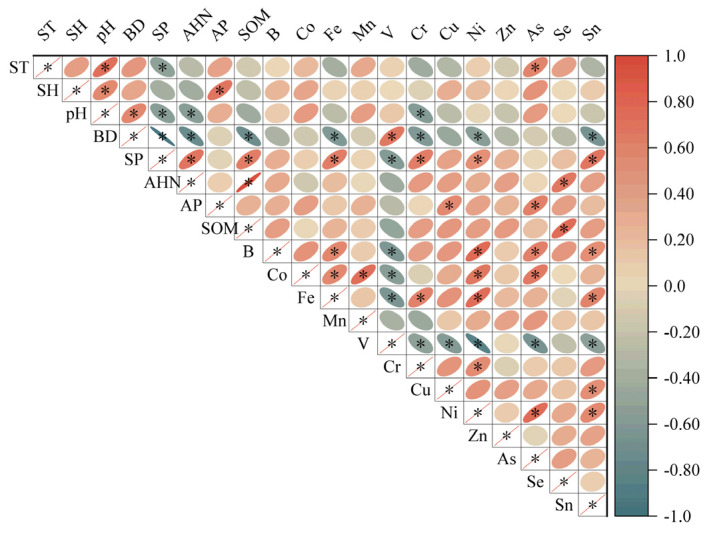
Pearson correlation between selected soil properties and soil minor and trace elements. Note: *, correlation is significant at the 0.05 level.

The Pearson correlation analysis indicated a significant relationship between minor and trace elements in the soil and various soil properties (Figure 4). The soil temperature showed significantly positive correlations with the concentrations of As, and pH was negatively correlated with Cr. The soil BD was significantly negatively correlated with the Fe, Cr, Ni and Sn concentrations, and porosity was correlated with V. In contrast, the soil total porosity was positively correlated with the concentrations of Fe, Cr, Ni and Sn and negatively correlated with V. Further, the soil Se contents were significantly positive correlations with the concentrations of AHN and SOM. The contents of AP were significantly positive correlations with the contents of Cu and As.

**Figure 5:**
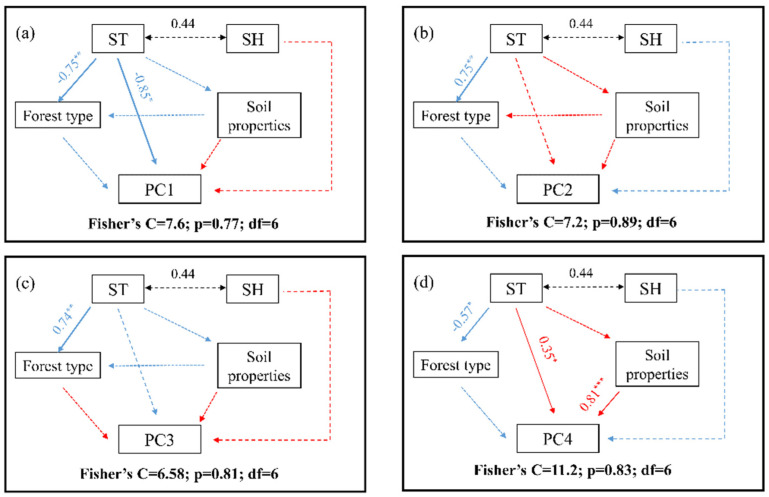
The influence of environmental factors on minor and trace elements in soil through structural equation modeling (SEM). (**a**) PC1 SEM plot, (**b**) PC2 SEM plot, (**c**) PC3 SEM plot, (**d**) PC4 SEM plot. Red solid arrows indicate positive effects, blue solid arrows indicate negative effects and dashed lines indicate non-significant paths. Significance levels of each predictor are * *p* < 0.05, ** *p* < 0.01, *** *p* < 0.001.

As the forest plots were close to each other and the annual mean temperature and annual precipitation conditions were close, we used soil temperature and soil humidity instead of temperature and precipitation for piecewise SEM analyses. The results obtained from SEM demonstrated the influence of climatic factors (ST and SH) and soil properties on each of the principal components related to the concentrations of trace elements. ST directly and negatively affects the forest type and PC1 (Figure 5a). PC4 was directly and positively influenced by ST and soil properties (Figure 5d). SH indirectly and negatively affects the PC2 and PC4 (Figure 5b,d) and positively and indirectly influences the PC1 and PC3 (Figure 5a,c). We did not observe a direct pathway for soil minor and trace elements effects of forest type in the model.

## 4. Discussion

### 4.1. Forest Type and Soil Depth Effect on Minor and Trace Elements in Soils

Soil has wide variations in minor and trace element concentrations due to soil proportions, vegetation type and bedrock weathering. Tree species can influence soil through various mechanisms, including the quantity and composition of litter, root activity, the microclimatic conditions they create and the ground vegetation that is established beneath their canopy [32]. The elements of B, Fe, Cr, Cu, Ni and Sn exhibited significant enrichment within the broadleaf forest and mixed broadleaf–conifer forest zones. Coniferous forests possess a high concentration of keratin, which serves to inhibit the adhesion and invasion of microorganisms on leaves that are abundant in keratin [33]. The rate of decomposition of leaves in coniferous forests is much smaller than that in broad-leaved forests. Broadleaf forests, such as *Larix principis-rupprechtii*, which thrive in cold climates characterized by high precipitation, exhibit the development of robust tracheids and fine root structures. This adaptation facilitates the formation of intricate and resilient root systems [34]. The resilient root systems facilitate the processes of rock fragmentation and soil weathering, thereby contributing to the release of essential nutrients, including Fe, Cu and other alkaline cations [35]. In contrast, the average concentrations of Co, Mn, V, Zn, As and Se were mainly enriched in coniferous forest zones. Coniferous forests demonstrate a greater capacity for soil acidification compared to broadleaf forests [35], resulting in enhanced leaching of cationic elements, including Zn, Mn and Co [36].

Our results show that average concentrations of Fe, Cr, Ni, Zn, As, Sn and Co increase with soil depth in BP forests. The metallic constituents present in the soil primarily originate from the weathering processes of the underlying bedrock [20]. The B concentrations present a trend of increasing then decreasing with soil depth; this phenomenon may be attributed to the presence of boron in the soil, which arises from the weathering of boron-containing minerals found in the underlying geological formations [37]. However, during the process of weathering, element B exhibits mobility and enters into solution, with its concentration being regulated by the presence of clay minerals, which can either adsorb or incorporate it [38], which results in the enrichment in B layers. We have observed that the concentrations of Se and Cu were higher in the A layer than the C layer, while the concentrations of these elements remained consistent across the study sites within the A horizon, indicating a lack of significant influence from the underlying bedrock [7]. Mn and V levels did not show differences between soil layers; thus, the stratification of soil may exhibit greater complexity, necessitating additional research to address the acceptable thresholds for this particular element.

### 4.2. Soil Minor and Trace Elements in Relation to Soil Properties and Climate

Temperature and precipitation represent the primary climatic variables that significantly affect plant survival and distribution [39,40]. Consequently, these factors contribute significantly to the spatial variability observed in extractable mineral elements [41,42]. Extreme temperatures, whether excessively low or high, can hinder plant growth and the functioning of soil microorganisms. This inhibition, in turn, limits the accumulation of organic matter and the absorption of minerals [43]. Precipitation also affects soil properties (e.g, SOM, AHN, AP), with increased SH due to precipitation increasing leaching along the soil profile, further affecting soil micronutrient distribution. The pH ranged from 6.34 to 7.01 (Table S2) and was generally weakly acidic, approaching neutrality. Research has indicated that the complexity and stability of microbial networks tend to decrease as the pH approaches neutrality. Moreover, research indicates that more complex microbial networks enhance both the efficiency and rate of ecosystem functioning relative to simpler networks, thereby facilitating increased leaching of cationic elements from the soil. Soil pH showed a significant positive correlation with SH and ST, and it can be hypothesized that SH and ST affect the accumulation of these elements in the soil by influencing the pH value. There was a significant negative correlation between soil BD and the concentration of Fe, Cr, Ni and Sn. This phenomenon can be ascribed to the strong correlation between biodiversity and various factors, including soil moisture, vegetation communities, soil texture and the content of organic matter [44,45]. Increases in bulk weight lead to poorer air permeability and less oxygen in the soil, limiting the activity of soil microorganisms and resulting in lower metal element concentrations. Se contents were significantly positive correlations with the concentrations of AHN and SOM. In general, the majority of Se present in soil is associated with organic matter, resulting in an increase in Se concentration corresponding to higher levels of SOM [46,47]. The concentration of As had significantly positive correlations with the contents of AP, as arsenic’s soil cycling and behavior are often linked to phosphorus [48]. We observed that Cu also had a strong significant correlation with AP. The complexation of copper by organic matter has been identified as the primary and most efficient mechanism for the retention of copper in soil environments [49], and the concentration of AP in the soil promotes the formation of copper complexes with soluble organic matter.

The SEM results showed that climate (ST and SH), forest type and soil properties have an effect on each of the principal components related to trace element concentrations. ST has a direct negative effect on variation in B, Fe, V, Cr and Ni concentrations. Several studies have shown that micronutrient availability is low under high-temperature conditions in semi-arid regions [50]. These findings help to explain the negative effect of temperature on B, Fe, V, Cr and Ni content in this study. The concentration of Se was directly and positively influenced by ST and soil properties. Selenium is classified as a chalcophile element, which makes it susceptible to secondary enrichment or dilution as a result of supergene geochemical processes [51]. Research indicates that the concentration of this substance in surface sediments is typically influenced by various geological and geographical factors, including the lithology of the bedrock, the types of vegetation present, climatic conditions, and elemental distributions [52,53,54]. In this study, we did not observe any direct pathway for soil minor and trace elements effects of forest type. This phenomenon can be ascribed to the dual origins of soil minor and trace elements, which include the erosion of local bedrock (lithogenic fraction) and contributions from atmospheric sources (atmospheric fraction) [7]. Little or no influence by forest type was observed.

## 5. Conclusions

In summary, the concentrations of B, Fe, Cr, Cu, Ni and Sn were mainly enriched in the broadleaf forest and mixed broadleaf–conifer forest zones. The average concentrations of Co, Mn, V, Zn, As and Se were mainly enriched in coniferous forest zones. We have observed that the concentrations of Se and Cu were higher in the A layer than the C layer, and Fe, Cr, Ni, Zn, As, Sn and Co concentrations increased with soil depth in BP forests. The B, Fe, V, Cr and Ni concentrations were directly and negatively affected by soil temperature, while the concentrations of Se are mainly influenced by soil temperature and soil properties.

The core area of the Winter Olympics is a hotspot for national ecological environmental protection and water conservation in the Beijing–Tianjin–Hebei region. Understanding the distribution of minor and trace elements in forest soils across the core area can provide important information on soil succession patterns in the boreal forest. Investigations of the principal drivers of elemental concentrations may help elucidate the response of elemental distributions to climate change.

## Data Availability

The data presented in this study are available on request from the corresponding author.

## Supplementary Materials

The following supporting information can be downloaded at https://www.mdpi.com/article/10.3390/biology14010082/s1, Table S1: Soil temperature and soil humidity of sampling sites (n = 9); Table S2: Variance analysis of soil chemical and physical properties between different forests and soil layers; Table S3: Descriptive statistics of minor and trace element concentrations in soils of the five forests.
