# Supplementary material for: Climate and Soil Properties Drive the Distribution of Minor and Trace Elements in Forest Soils of the Winter Olympic Core Area
Source: Biology (Basel). 2025 Jan 16;14(1):82. doi: 10.3390/biology14010082 (PMC11763068; doi:10.3390/biology14010082)
Supplement: Supplementary file 1 [file biology-14-00082-s001.zip › biology-3381603-supplementary.pdf]

Table S1. Soil temperature and soil humidity of sampling sites (n=9).

Forest	Month	Soil temperature (℃)	Soil humidity (%)
PS	July	15.9	60.1
	October	15.2	59.4
	January	-8.5	44.0
	Apr	16.4	44.8
BP	July	13.7	56.0
	October	6.0	43.1
	January	-8.0	24.3
	Apr	5.0	34.4
LP	July	16.9	61.9
	October	12.6	54.4
	January	-9.0	46.3
	Apr	5.6	55.1
PA	July	21.3	66.4
	October	15.4	54.2
	January	-13.0	33.3
	Apr	22.0	41.0
MF	July	22.3	75.3
	October	15.1	59.4
	January	-12.0	30.7
	Apr	10.7	31.0

Table S2. Variance analysis of soil chemical and physical properties between different forests and soil layers

Forest	Horizon	$pH_{(H_2O)}$	Bulk density (g/cm^3)	Soil porosity (%)	SOM(g/kg)	AHN (mg/kg)	AP (mg/kg)
PS	A	6.82±0.07Aa	1.02±0.07ABa	49.68±7.63ABa	71.6±8.09Aa	299.69±89.09Aa	8.30±1.68Aa
	B	6.91±0.03Aa	1.12±0.07ABa	46.31±4.91ABa	77.7±12.63Aa	298.15±112.93Aa	9.82±1.77Aa
	C	6.95±0.15Aa	1.12±0.13ABa	46.36±5.47ABa	67.1±9.62Aa	230.81±92.68Aa	8.88±2.45Aa
BP	A	6.74±0.05Aa	0.85±0.08Aa	59.10±3.80Aa	70.0±2.51Aa	256.49±22.51Aa	9.02±1.22ABa
	B	6.65±0.06Aa	1.02±0.04Aa	50.89±0.98Aab	67.0±2.40Aa	249.86±21.40Aa	9.45±0.60ABa
	C	6.72±0.14Aa	1.28±0.23Ab	42.12±9.63Ab	49.0±6.65Ab	165.49±58.65Ab	11.59±4.24ABa
LP	A	6.83±0.16Aa	1.09±0.30ABa	46.81±11.99ABa	53.7±3.41Ba	158.13±7.51Ba	11.68±3.78Ba
	B	7.00±0.14Aa	1.12±0.04ABa	46.12±2.07ABa	47.3±8.44Ba	149.18±16.74Ba	7.84±2.01Bb
	C	7.01±0.09Aa	1.32±0.13ABa	41.65±4.97ABa	40.0±3.21Bb	100.88±32.61Bb	8.76±1.13Bab
PA	A	6.84±0.15Aa	1.25±0.08Ba	42.87±9.45Ba	45.9±3.17Ba	136.60±37.77Ba	10.66±2.12ABa
	B	6.91±0.08Aab	1.40±0.03Bab	38.38±8.68Ba	31.4±2.40Bb	80.05±28.40Bb	5.45±1.58ABb
	C	7.00±0.08Ab	1.65±0.18Bb	31.80±9.04Ba	13.0±2.20Bc	35.96±3.20Bc	4.31±0.80ABb
MF	A	6.40±0.23Ba	0.79±0.02Aa	61.58±17.12Aa	85.9±7.41Ca	393.58±60.57Ca	3.59±1.22Ca
	B	6.63±0.19Bb	0.87±0.08Aa	57.52±1.34Ab	68.2±3.67Cb	294.91±57.02Cb	9.26±1.13Cb
	C	6.34±0.05Ba	1.13±0.08Ab	47.47±5.05Ac	43.2±5.01Cc	188.92±76.01Cc	2.83±0.52Ca

Different uppercase letters indicate significant differences between soil types within the same soil layer ($P < 0.05$), and different lowercase letters indicate significant differences among soil layers within the soil types ($P < 0.05$). SOMis Soil organic matter, AHN is alkali-hydrolyzable nitrogen, AP is available phosphorus.

Table S3. Descriptive statistics of minor and trace element concentrations in soils of the five forests.

	B (mg/kg)	Co (mg/kg)	Fe (g/kg)	Mn (mg/kg)	V (mg/kg)	Cr (mg/kg)	Cu (mg/kg)	Ni (mg/kg)	Zn (mg/kg)	As (mg/kg)	Se (mg/kg)	Sn (mg/kg)
**PS**												
Mean	31.44	16.66	34.32	652.33	12.36	60.17	20.17	26.44	60.61	10.21	0.20	2.01
Min	27.20	15.70	31.90	605.00	11.80	57.00	18.00	25.00	54.00	9.36	0.15	1.47
Max	36.70	18.70	37.80	682.00	13.10	66.00	22.00	29.00	74.00	10.70	0.31	2.79
Standard deviation	2.66	0.73	1.42	19.17	0.42	2.38	1.65	1.10	5.76	0.38	0.05	0.43
Coefficientof Variation (%)	8	4	4	3	3	4	8	4	1	4	27	21
**BP**												
Mean	32.50	16.54	37.22	573.28	11.93	72.89	24.72	27.06	60.72	9.94	0.15	2.63
Min	26.90	15.50	32.90	523.00	10.50	63.00	19.00	24.00	52.00	8.47	0.11	1.63
Max	35.50	18.10	41.90	633.00	12.80	99.00	50.00	32.00	79.00	10.80	0.18	4.36
Standard deviation	2.38	0.87	2.68	30.00	0.67	10.01	9.23	2.41	7.41	0.70	0.02	0.84
Coefficientof Variation (%)	7	5	7	5	6	14	37	9	12	7	14	32
**LP**												
Mean	30.21	17.53	35.82	720.17	12.55	54.72	21.39	26.72	63.39	10.16	0.14	2.32
Min	25.70	16.30	33.20	621.00	11.90	34.00	18.00	24.00	59.00	9.64	0.10	1.62
Max	35.40	18.60	39.30	772.00	13.20	68.00	25.00	29.00	77.00	11.10	0.20	3.01
Standard deviation	2.20	0.67	1.79	46.53	0.40	14.07	2.35	1.49	5.29	0.45	0.03	0.46
Coefficientof Variation (%)	7	4	5	6	3	26	11	6	8	4	20	20

**PA**												
Mean	28.52	15.85	32.87	577.44	16.89	54.61	17.28	23.39	56.89	9.25	0.12	1.55
Min	16.90	11.50	24.90	416.00	12.00	30.00	8.00	12.00	49.00	5.11	0.05	1.16
Max	33.60	17.50	36.70	630.00	36.00	64.00	20.00	27.00	68.00	11.10	0.24	2.24
Standard deviation	4.64	1.65	3.37	63.44	7.05	9.94	3.66	4.41	6.15	1.60	0.05	0.25
Coefficientof Variation (%)	16	10	10	11	42	18	21	19	11	17	42	16
**MF**												
Mean	30.24	16.18	36.58	614.00	12.33	67.89	21.00	25.89	61.67	9.13	0.14	2.27
Min	26.80	14.60	33.00	574.00	11.90	62.00	19.00	23.00	58.00	8.46	0.08	1.74
Max	34.90	17.80	39.10	684.00	12.80	74.00	24.00	29.00	68.00	10.30	0.20	3.49
Standard deviation	2.59	0.81	1.79	33.50	0.31	3.86	1.71	1.60	3.27	0.51	0.04	0.46
Coefficientof Variation (%)	9	5	5	5	3	6	8	6	5	6	27	20
